# Comparison of the Impact of Conventional and Web-Based Pulmonary Rehabilitation on Physical Activity in Patients With Chronic Obstructive Pulmonary Disease: Exploratory Feasibility Study

**DOI:** 10.2196/28875

**Published:** 2022-03-10

**Authors:** Emma Chaplin, Amy Barnes, Chris Newby, Linzy Houchen-Wolloff, Sally J Singh

**Affiliations:** 1 Centre for Exercise and Rehabilitation Science National Institute for Health Research Leicester Biomedical Research Centre- Respiratory University Hospitals of Leicester National Health Service Trust Leicester United Kingdom; 2 School of Medicine University of Nottingham Nottingham United Kingdom; 3 Respiratory Sciences University of Leicester Leicester United Kingdom

**Keywords:** SPACE for COPD, internet, web-based, chronic obstructive pulmonary disease, pulmonary rehabilitation, physical activity, exercise, chronic disease, COPD, rehabilitation

## Abstract

**Background:**

Pulmonary Rehabilitation (PR) increases exercise capacity, with less clear evidence regarding physical activity (PA). The World Health Organization recommends at least 150-300 minutes of moderate-intensity or 75-150 minutes of vigorous-intensity aerobic PA per week to reduce the risks of chronic disease.

**Objective:**

The objective of this study was to assess the effectiveness of conventional PR versus web-based PR with respect to changes in PA.

**Methods:**

Patients with COPD were randomized to either conventional PR classes (n=51) or a web-based PR program (n=52) for 7 weeks in a feasibility study. Accelerometers (Sensewear) were worn before and after the intervention, and PA was measured as steps per day and mean bouts of moderate activity for ≥2, ≥5, ≥10, and ≥20 minutes. Measures were derived for patients with ≥8 hours of data per day for ≥4 days, using the R package for statistical analysis. Variables were explored to examine their relationships with bouts of activity.

**Results:**

Baseline characteristics did not differ significantly between groups. Complete PA data were available for the groups receiving web-based (n=20) and conventional (n=34) PR interventions. The web-based PR group demonstrated a nonsignificant increase in the number of steps per day, which mainly comprised short bouts of moderate to vigorous intensity PA when compared to the conventional PR group (*P*=.20). The conventional PR group demonstrated increased 20-minute bouts of PA by 49.1%, although this was not significant (*P*=.07). At baseline, age (*r*=–0.21, *P*=.04), BMI (*r*=–0.311, *P*=.004), and FEV_1_ (forced expiratory volume in 1 second; % predicted; *r*=–0.248, *P*=.048) were significantly correlated with 10-minute bouts of PA; however, this was not observed post intervention.

**Conclusions:**

The analysis revealed a nonsignificant difference in the pattern of PA between groups receiving conventional vs web-based PR—the former being associated with an increase in 20-minute bouts, while the latter having demonstrated an increase in the number of steps per day. There appears to be a differing response emerging between the two interventions.

**Trial Registration:**

International Clinical Trials Registry ISRCTN03142263; https://tinyurl.com/y4dmfyrb

## Introduction

### Background

Individuals with chronic obstructive pulmonary disease (COPD) have poor exercise capacity and low physical activity (PA) levels [1], which are associated with an increased risk of hospital admission, poor quality of life, and increased mortality [2]. While pulmonary rehabilitation (PR) focuses on improving functional exercise capacity, this does not necessarily translate into increasing PA, the latter defined by the World Health Organization as “any bodily movement produced by skeletal muscles that requires energy expenditure” [3]. A recent study attempted to increase the amount of time patients were physically active by using pedometers as an adjunct to PR. However, the addition of step count targets during a PR program did not improve moderate-intensity PA levels [4].

PA is considered a modifiable risk factor for morbidity and mortality in people with COPD and those with other long-term conditions [2]. Although there are known benefits of pulmonary rehabilitation (PR) in terms of exercise capacity, psychological functioning, and quality of life, a recent review showed poor evidence about determinants of PA, including the impact of treatment or interventions in people with COPD [5]. National guidelines recommend that older adults should accumulate 150-300 minutes of moderate-intensity or 75-150 minutes of vigorous-intensity aerobic PA per week [6]. Achieving these targets is difficult when exercise capacity is reduced owing to shortness of breath and reduced muscle strength. In addition, the availability and access to pulmonary rehabilitation programs in the United Kingdom is limited, and attrition rates are often high [7]. To address this issue, there is an increasing appreciation among clinicians to offer wider choice in the delivery of rehabilitation.

Home-based rehabilitation has recently been shown to be an alternative to center-based PR. Grosbois et al [8] have shown home-based PR consisting of unsupervised physical exercises, therapeutic patient education, and self-management to be effective in the short, medium (6 months), and long term (12 months) at improving exercise capacity, mood, and quality of life [8]. Furthermore, an internet-based walking program for patients with COPD, which focused exclusively on step counts, increased the daily number of steps by >1000 over 3 months [9], and a randomized controlled trial of a pedometer-based program versus a standard program of PA encouragement alone increased step counts by 3080 (SD 3254) compared to 138.3 (SD 1950), respectively [10].

The effectiveness of PA interventions is determined by an improvement of >600 steps per day [11]. The evidence for home- or web-based PR to increase PA, however, is less well-established in COPD but has been demonstrated to be effective in cardiac rehabilitation [12]. A recent home-based PR trial has revealed a reduction in the amount of time during which all patients are sedentary (mean change -44 minutes) and an increase in the amount of time patients are performing bouts of moderate to vigorous PA (mean change 16 mins) following a home program [13]. However, there were no significant between group differences. Within our department, a *Self-Management Programme of Activity, Coping and Education*, “*SPACE for COPD*” has been developed (manual version) [14]. This program has been shown to be effective in primary care [15], where there were significant between-group differences in steps at 6 weeks in favor of the home training program and was recently shown to also be comparable to conventional rehabilitation in improving exercise performance and perceived dyspnea [16]. We have since developed a web-based provision of this program, SPACE for COPD. The protocol for the interactive web-based feasibility trial has been published previously [17]. Details of the nonclinical feasibility data and primary outcome of the main study are reported in a separate publication [18]. Significant within-group changes were observed in exercise capacity and quality of life, but there were no significant differences between groups. The study utilizes the pre-existing SPACE for COPD manual but in a web-based format. In brief, the study identified an improvement in both quality of life and endurance walking times.

### Aims

The purpose of this secondary exploratory analysis was to compare the impact of the two interventions on PA with respect to bouts of total activity and to determine whether the response to the center-supervised and remotely supervised program differed in terms of the individuals’ PA profiles and responses to an exercise training program. The relationship between PA and routinely collected clinical data at baseline and after the intervention was also explored.

## Methods

### Ethics Approval

Participants were recruited between 2013 and 2015, and ethical approval was granted by Northampton Ethics Committee of the UK National Research Ethics Service (12/EM/0351). All study participants signed an informed consent form prior to their enrollment. Individual patients could not be identified through the information presented in this analysis.

### Eligibility Criteria

Participants were eligible to partake if they had a confirmed diagnosis of COPD, defined as having a postbronchodilator FEV_1_ (forced expiratory volume in 1 second) of <80% and a predicted FEV_1_ forced vital capacity ratio of ≤0.70 (GOLD stage 2-4) and a Medical Research Council Dyspnoea Scale score of 2-5. Patients had to be willing to partake in either arm of the study. Patients were required to have had access to the internet for more than 3 months, the ability to navigate a variety of websites (eg, uses e-shopping or e-banking websites), and use email regularly. Patients also had to be able to read and write in English.

Patients were excluded if they were unable to participate in the exercise component of the rehabilitation program owing to other comorbidities or had undergone PR in the previous 12 months.

### Randomization

Randomization was performed using a web-based program [19]. Participants were allocated on a 1:1 ratio to either a standard care (conventional PR program) or an intervention group (web-based PR program).

### Trial Interventions

#### Intervention Group: Web-Based PR Program

Following randomization to the intervention group, the participants attended a standardized introductory session where they were provided a password-protected secure log-in to the website as well as written instructions on website navigation. There are 4 stages to the program, each with a number of mandatory tasks to complete before moving onto the next task or stage. A description of the different stages is provided in Textbox 1. Upon completion of an information needs questionnaire at registration, gaps in knowledge were identified, and patients were signposted to relevant educational topics. Participant’s progress was monitored and reviewed on the internet regularly and through weekly contact with a health care professional. As in conventional PR, patients were encouraged to exercise daily at home and record their progress in the web-based exercise diary section. The exercise program consisted of both aerobic and strength training. Patients were advised to walk at the pace that was determined from the baseline maximal exercise walking tests performed in the initial assessment, increasing the amount of time they walked for each day. Strength training comprised both upper and lower limb exercises using hand-held weights. Both exercise components progressed while maintaining a visual analogue scale (VAS) rating of 4-7. It was anticipated from previous work [20] that it would take approximately 6-8 weeks to work through the web-based program.

**Stages of the web-based pulmonary rehabilitation program.**

**Stages:**

- Stage 1: introduction to exercising and goal setting, exercise safety quiz, and reading educational material
- Stage 2: introduction to the aerobic exercise program, setting walking targets, and reading educational material
- Stage 3: introduction to the strength training program, setting strength targets, continuing aerobic training, and reading education material
- Stage 4: maintaining strength and aerobic training, reviewing educational material, and a knowledge quiz

#### Standard Care Group: Conventional PR Program

Patients randomized to standard care commenced conventional rehabilitation, as described by the British Thoracic Society’s guidelines [21], in accordance with the standard care at their referred site, which was either hospital- or community-based. The hospital-based program was of 7 weeks (4 weeks supervised and 3 weeks unsupervised) in total. Any sessions that were missed could be completed later because it was a rolling program. In the community-based programs, patients could attend a maximum of 12 sessions within the closed program.

Conventional PR programs at either referral site consisted of 2 weekly sessions, each lasting 2 hours, which were divided into an hour for exercise training, consisting of both aerobic and resistance training, and an hour for an education session covering a variety of relevant self-management topics.

The trial interventions for both the web-based and conventional pulmonary rehabilitation groups have previously been described in detail [18].

### Physical Activity

All participants wore a Bodymedia Sensewear triaxial accelerometer (APC Cardiovascular). Algorithms within the software convert the data to produce meaningful outcome variables, which include the number of steps, energy expenditure in metabolic equivalence to tasks (METs): a multiple of the resting rate of oxygen consumption per minute (one MET is equal to that of the O_2_ consumption at rest, which is approximately 3.5 mL/kg/minute) and PA duration (vigorous >6, moderate 3-6 METs, and light >1.5 METs intensity).

Accelerometer data were collected for 7 days at baseline and a further 7 days following discharge. None of the data were collected while the patients were participating in either intervention. Measures were derived for patients with ≥8 hours of data per day for ≥4 days [22] at each time point using the R package for statistical analysis [23]. The Sensewear accelerometer has been previously validated in COPD [24,25], and 4 days was proven sufficient to demonstrate treatment effects.

### Data Analysis

#### Sample Size

Owing to the original study being a feasibility study, a formal sample size calculation was not required to detect between-group changes. We aimed to recruit around 100 patients within the timeframe of the operational phase of the trial. This was based on previous studies carried out in the PR service and deemed a reasonable number to assess the recruitment or retention rate and inform the planning of a subsequent randomized controlled trial. This is in line with recommendations by Lancaster et al [26] on the number of participants required in a feasibility study to estimate a parameter. Furthermore, in a recent audit of feasibility studies in the United Kingdom, it was found that a median sample size for a 2-arm trial was 36 and 30 per arm, respectively, for dichotomous and continuous endpoints [27]. Although the data in this exploratory study, based on secondary and per protocol analysis, fell slightly below this in the number of participants in the web-based PR group (n=20), the data from the original study were collected for 103 participants (web-based care, n=51; usual care, n=52).

#### Statistical Analysis

Baseline characteristics were compared between groups using a 2-tailed independent samples *t* test. A 2-tailed paired samples *t* test was used to compare within-group changes, and a 2-way repeated measures analysis of variance (ANOVA) was used to compare the differences between the two treatment groups in the number of steps and PA pattern at the two time points. All *t* tests, repeated measures ANOVA, and factor analysis were performed using the SPSS (version 22; SPSS Inc) with a level of significance set at *P*<.05. The change in time in bouts (2-20 minutes) expressed as a percentage change, the mean change in bout length of moderate to vigorous PA (MVPA), daily MET level, and percentage time in moderate activity were explored.

Correlations between routinely collected clinical data and PA were explored using the Spearman rank correlation coefficient.

### Patient and Public Involvement

A preprotocol award from the National Institute of Health Research (NIHR) East Midlands Research Design Service enabled us to conduct a focus group with current and ex-PR patients to gain feedback on the prototype website, with particular regard to features that would increase the interactivity and usability for service users as well as addressing any concerns such as data security. The website has undergone practical “road-testing” by members of the focus group and other members of the departmental patient and public involvement (PPI) group to ensure that participants can access the website and navigate the site easily. A member of the PPI group attended the study and steering group meetings, and a strategy for disseminating the results was thus coordinated.

## Results

### Results Overview

**Figure 1:**
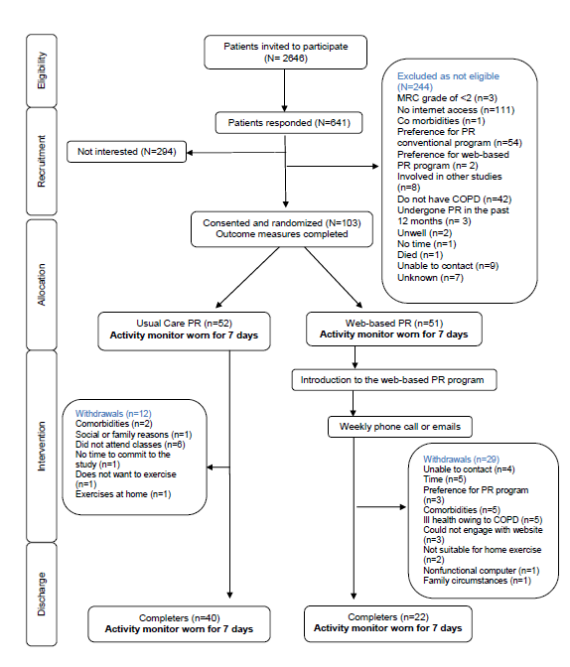
CONSORT (Consolidated Standards of Reporting Trials) flow diagram of participation. COPD: chronic obstructive pulmonary disease, MRC: Medical Research Council, PR: pulmonary rehabilitation.

The flow of eligibility, screening, randomization, and follow-up in the study is shown in Figure 1. The baseline characteristics of participants with complete PA data are shown in Table 1. There were no significant differences in age, BMI, FEV_1_, smoking status, and home oxygen usage between the web-based and conventional PR groups. Exercise capacity at baseline was similar in both groups. For participants assigned to the web-based PR group, the mean number of weeks to complete the program was 11.5 (SD 4.1), and the mean stage reached for those in the web-based PR group, who withdrew from the intervention, was stage 3 (IQR 1-4). The total number of complete accelerometer data sets for PA was 34 for the conventional PR group and 20 for the web-based PR group.

**Table 1:** Baseline participant characteristics (N=54).

Characteristics		Web-based pulmonary rehabilitation group (n=20)	Conventional pulmonary rehabilitation group (n=34)	Between-group differences, *P* value	
Gender (males/females), n		18/2	19/15	.009^a^	
Age (years), mean (SD)		68.3 (6.5)	67.4 (8.6)	.60	
BMI (kg/m^2^), mean (SD)		27.2 (5.5)	29.8 (6.6)	.13	
Forced expiratory volume in 1 second (L), mean (SD)		1.52 (0.7)	1.47 (0.6)	.84	
Forced expiratory volume in 1 second (% predicted), mean (SD)		54.2 (26.9)	55.8 (19.4)	>.99	
**Smoking status: current, n (%)**		3 (15)	3 (8.8)	.92	
	Nonsmoker	0 (0)	3 (8.8)		
	Ex-smoker	17 (85)	26 (76.5)		
	Unknown	0 (0)	2 (5.9)		
**Home oxygen usage, n (%)**					.83
	Yes	4 (20)	6 (17.6)		
	No	16 (80)	28 (82.4)		
Medical Research Council Dyspnoea Scale score, median (IQR)		3 (2-4)	3 (2-4)	.62	
**Medical Research Council grade, n (%)**					.62
	2	9 (45)	15 (45.5)		
	3	5 (25)	8 (24.2)		
	4	5 (25)	10 (30.3)		
	5	1 (5)	0 (0)		
Incremental shuttle walking test (m), mean (SD)		338.5 (185.7)	286.8 (159.4)	.28	
Endurance shuttle walk test (seconds), mean (SD)		263.9 (250.1)	256.2 (157.1)	.89	

^a^Significant at *P*<.05 between groups.

### Number of Steps Per Day

**Figure 2:**
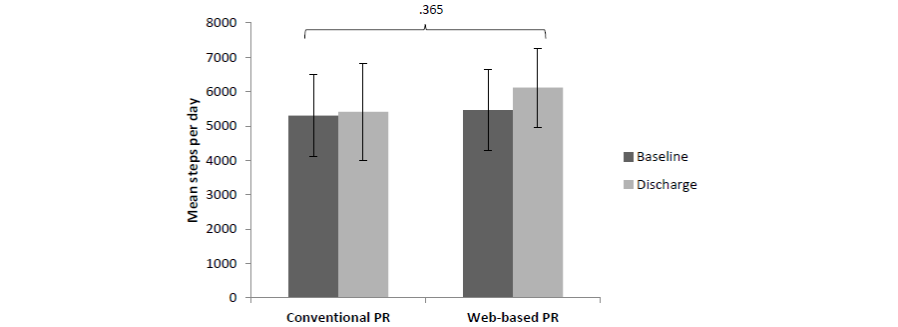
Comparison of the mean number of steps per day during waking hours between the conventional and web-based pulmonary rehabilitation groups at baseline and post intervention. PR: pulmonary rehabilitation.

There were no significant differences in PA, in terms of steps per day, between the groups at baseline (*P*=.86). There was a nonsignificant increase (*P*=.20) in the number of steps per day from 5465 to 6112 (12%) in the web-based PR group compared with the conventional PR group (*P*=.80; n=5300-5409, 2%; Figure 2).

### PA Pattern of Accumulation

**Figure 3:**
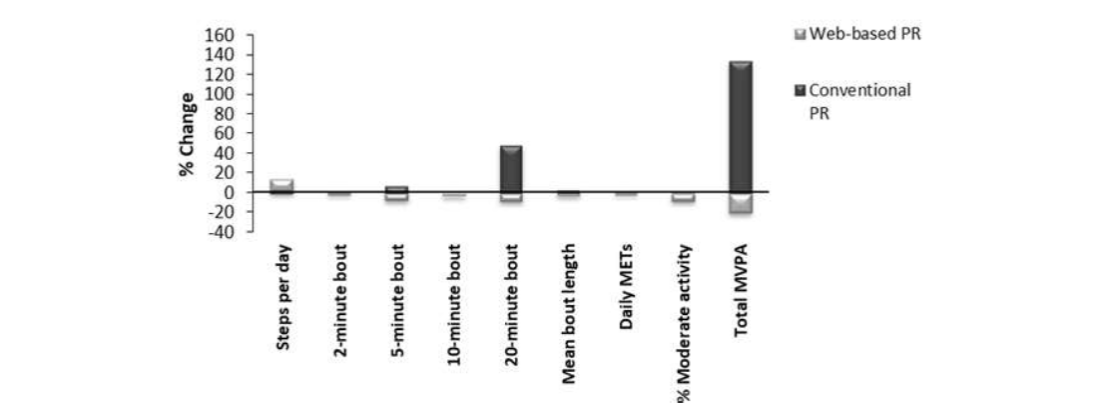
Comparison of the percentage change in physical activity between the conventional and web-based pulmonary rehabilitation groups. MET: metabolic equivalence to task, MVPA: moderate to vigorous physical activity, PR: pulmonary rehabilitation.

The increase in the number of steps observed in the web-based PR group was accumulated mainly through an increase of 2-minute bouts of PA (Figure 3). In contrast, the conventional PR group displayed increased 20-minute bouts of PA by 49%, although this was not significant (*P*=.07). The mean bout length of PA was similar between both groups (2.7-2.8 minutes) and did not significantly change following either intervention. Although the percentage of time in moderate activity was greater in the web-based PR group than in the conventional PR group (9.43 vs 8.14, respectively), this was not increased post intervention. Daily METs were similar in both groups, with those in the web-based PR group increasing only slightly at discharge (Table 2).

**Table 2:** Physical activity pattern before and after the intervention.

Activity pattern	Web-based pulmonary rehabilitation group (n=20)		Conventional pulmonary rehabilitation group (n=34)	
	Baseline, mean (SD)	Discharge, mean (SD)	Baseline, mean (SD)	Discharge, mean (SD)
Steps per day	5464.6 (3013.3)	6111.7 (2464.2)	5300.1 (3402.7)	5409.4 (3377.7)
Daily metabolic equivalence to tasks	1.52 (0.3)	1.54 (0.2)	1.44 (0.4)	1.42 (0.4)
Percentage of moderate activity	10.25 (8.8)	9.43 (4.2)	8.07 (5.9)	8.14 (7.3)
Mean bout length	2.8 (0.8)	2.7 (0.7)	2.7 (0.9)	2.8 (1.0)
Number of 2-minute bouts	21.1 (18.5)	21.4 (8.9)	18 (12.7)	17.9 (14.6)
Number of 5-minute bouts	7.2 (8.5)	6.7 (4.0)	5.5 (4.8)	5.9 (6.0)
Number of 10-minute bouts	2.11 (2.8)	2.1 (1.8)	1.6 (2.1)	1.6 (1.9)
Number of 20-minute bout	0.47 (0.7)	0.43 (0.4)	0.26 (0.4)	0.38 (0.5)
Total moderate to vigorous physical activity	82.03 (69.9)	65.7 (39.7)	102.9 (78.5)	241.1 (69.4)

### Correlation Between Clinical Data and PA

As there were no significant differences between groups, the groups were collapsed and correlations between routinely collected clinical data and PA were explored. The variables age (*r*=–0.21, *P*=.04), BMI (*r*=–0.311, *P*=.004), and FEV_1_ % predicted (*r*=–0.248, *P*=.048) significantly correlated with 10-minute bouts of PA at baseline. This effect was eliminated post intervention for age and FEV_1_, but not for BMI (Table 3).

**Table 3:** Correlations between age, BMI, and FEV_1_ (forced expiratory volume in 1 second) and physical activity before and after the intervention.

Variables	Preintervention		Postintervention	
	*r*	*P* value	*r*	*P* value
Age	–0.21	.04^a^	0.037	.78
BMI	–0.311	.004^a^	–0.449	<.001^a^
FEV_1_ (forced expiratory volume in 1 second; % predicted)	–0.248	.048^a^	–0.034	.84

^a^Significant at *P*<.05.

## Discussion

### Principal Findings

One of the main outcomes of the European Respiratory Society task force on PA in COPD was to understand how improvements in exercise capacity, dyspnea, and self-efficacy following PR might translate into PA [28]. PR is well known to improve exercise capacity and quality of life, but the data are inconsistent for PA [28-30]. This may be a consequence of heterogeneity of interventions and measurements of PA, making it difficult to compare studies [31], or in fact suggests that the traditional focus of PR programs is on improving functional capacity, not necessarily PA.

The results from this study show that web-based PR increased the number of steps (Figure 2) by 12%; although this was not significant, it is most likely a reflection of the small sample size. The number of steps increased by 647 in the web group, in line with the suggested Minimal Clinically Important Difference for pedometer steps in COPD, estimated at 600-1181 steps [11]. Further analysis showed that the increased step count in the web-based PR group mainly comprised 2-minute bouts of PA (Figure 3), with very few 5-, 10-, and 20-minute bouts of PA. On the other hand, participants in the conventional PR program showed a trend to increase 20-minute bouts of activity, but this was not reflected in the overall step count.

Although the time spent in moderate-intensity PA was greater in the web-based PR group than in the conventional PR group, this did not translate into an increase in the total amount of MVPA. The pattern of PA is more sporadic in the web-based PR group, whereas the conventional PR group elicits a change through more prolonged bouts of PA (Figure 3). Participants in the conventional PR group were able to increase their 20-minute bouts of moderate PA by 49.1% (Figure 3); although not significant, this may be clinically meaningful. This may suggest that a more supervised approach is needed to achieve longer bouts of PA at the level of ≥3 METs. These data are interesting and suggest that although the increase in steps is a potentially positive outcome of an intervention, the web-based PR participants did not as a group improve their prolonged bouts of activity as was observed and anticipated in the conventional PR group. These data suggest that for this population, to improve exercise behaviors (ie, prolonged bouts of MVPA), supervision is required. In comparison to this, a study using a smartphone-based PA telecoaching approach [32] found that patients requiring more contact from health care professionals experienced less PA benefits. However, patients in our study do appear to have increased their overall PA in the absence of any supervision, and this translated to a significant change in endurance walking times, which was seen in both groups. This is in line with a study by Demeyer et al [33], which showed that a 12-week semiautomated telecoaching intervention, which included an exercise booklet and step counter, significantly increased the amount (29% from baseline in terms of steps per day) and intensity of PA in patients with COPD. In comparison, our study also showed an increase, although small, in the number of steps per day of 12% in the web-based PR group from baseline. The physiological benefits gained from interval training have been shown to translate into clinically meaningful improvements in daily activity levels [34]. Louvaris et al [34] reported a 27% increase in the number of steps per day in the interval training group, which remained significantly greater 12 weeks following completion of PR, suggesting that this mode of training may be better to impact activities of daily living. A recent review challenges the relevance of PA patterns in patients with COPD, stating which is more important, “more time spent in higher intensity PA or less time spent in a sedentary state?” [31].

The American Thoracic Society/European Respiratory Society policy statement recommends alternative approaches—for example, step counters or telerehabilitation—may be best placed as a maintenance strategy for PR [35]. Using these strategies have not only shown to increase patients step counts and PA but also reduced the risk of exacerbations and hospital admissions [36].

Factors associated with PA have largely been cross-sectional, and from our data, moderate correlations in PA show a trend with respect to age, BMI, and FEV_1_ (% predicted) at baseline (Table 2), which is consistent with the existing literature [37-39]. There is a lack of data examining the direction of association and limited postintervention data describing these associations.

It is interesting to note that post intervention, the programs appear to have overcome the negative association between age and FEV_1_ on PA but not on BMI, which has a more significant correlation at baseline. This suggests that rehabilitation programs can potentially reverse the negative impact of FEV_1_ and age on PA, but this requires further exploration.

BMI remains highly significantly associated after PR, suggesting that in a population with obesity, additional interventions may be required to influence BMI. These data and those from previous studies, where BMI was used as a prognostic measure in COPD, have shown that both PR and PA have no influence on BMI, and as a result, a PR program was shown to be effective across the BMI spectrum; therefore, it is recommended that patients are referred irrespective of their BMI [40]. This may also be true for PA improvements.

Other studies have found factors such as respiratory and metabolic variations to be associated with PA [41]. Interestingly these changes did not differ across the GOLD stages. When attempting to stratify patients, which may improve in their PA post PR, exercise tolerance was found to be the strongest baseline independent factor to predict an improvement in PA [42]. In this study, it appears that those who gained more in terms of number of steps had a higher exercise capacity at baseline, although this was not true for those who increased their MVPA overall.

### Limitations

The main limitation of this study is that it is an exploratory analysis and is based on secondary analysis, and, per protocol, had a small sample size. Furthermore, there is a high risk of bias and a risk of overestimating any likely effect since this study only performed per protocol analysis. This was a highly selected group as the patient’s needed to be web literate and willing to follow the web-based program; therefore, this selection bias may limit external validity. There was also a high withdrawal rate from the web-based PR group, which is an important limitation when interpreting the results. This was mainly due to challenges experienced around a technology-based intervention. Loeckx et al [32] reported that approximately 8% of patients reported difficulty using technology. In this study, it was found that the exercise component of the web-based program was difficult, but once it was simplified after obtaining patient feedback, completion rates improved. There were no significant differences between the groups even though more participants withdrew from the web-based PR group. A previous study in 2010 [43] suggested that levels of daily activity may be vulnerable to seasonal variations. The progression of physical inactivity in patients with COPD has also been studied with respect to climate conditions (eg, temperature, day length, and rainfall) [44]. A significant decrease in PA was seen over a period of 1 year, which was further affected by the hours of rainfall. Activity monitors were worn in our study during different time points of the year, depending on recruitment; therefore, seasonal variation may also have been a factor influencing PA. Nevertheless, this is a novel exploration of 2 interventions for individuals with COPD, which appear to have different effects based on the level of supervision.

### Clinical Implications

When advising patients to increase their PA, promoting either multiple short bouts or long single bouts may be equally beneficial. Alternative approaches to increase PA may be more beneficial as a maintenance strategy.

### Conclusions

The combination of a highly selected group of participants and the exploratory analysis approach renders it difficult to make generalizations. However, there was a nonsignificant difference in the pattern of PA between conventional and web-based PR groups. Conventional PR was associated with an increase in 20-minute bouts of PA. Effects of age and FEV_1_ on PA can be overcome by taking part in rehabilitation, but BMI remains unaffected. This study shows a novel analysis of PA data, which could potentially be used as part of stratifying interventions based on measurements of PA and exercise capacity for individuals with COPD [45]. The data show that focusing on the number of steps alone can result in missing important messages about the pattern of PA.

## Abbreviations

COPD: chronic obstructive pulmonary disease
FEV_1_: forced expiratory volume in 1 second
MET: metabolic equivalence to task
MVPA: moderate to vigorous physical activity
NIHR: National Institute of Health Research
PA: physical activity
PPI: patient and public involvement
PR: pulmonary rehabilitation
SPACE for COPD: Self-Management Programme of Activity, Coping and Education for chronic obstructive pulmonary disease
